# Thermodynamic Origin of the Linear Pressure Dependence of DNA Thermal Stability

**DOI:** 10.1021/acs.jpclett.4c01563

**Published:** 2024-08-28

**Authors:** Jurij Lah, San Hadži

**Affiliations:** Faculty of Chemistry and Chemical technology, University of Ljubljana, Večna pot 113, 1000 Ljubljana, Slovenia

## Abstract

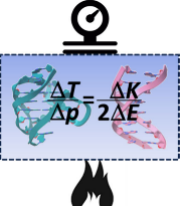

High pressure affects the structure and function of DNA and is a key parameter for studying the origin and physical limits of life. Different types of DNA structures systematically show a linear pressure dependence of thermal stability (up to ∼200 MPa), which is maintained even when the solution composition is changed. The reasons behind the linear pressure dependence are not understood. We have performed a thermodynamic analysis of the pressure-, temperature- and composition-dependent (un)folding of various polynucleotide duplexes and G-quadruplexes. We demonstrate that the reason for the observed linearity is the link between compressibility and expansibility, both of which largely depend on DNA hydration. We predicted the temperature and pressure dependence of compressibility and expansibility of (un)folding and explain how they affect the corresponding volume change and thermodynamic stability parameters. These predictions indicate the existence of a convergence temperature at which compressibility and volume of (un)folding simultaneously become equal to zero.

In native state biomacromolecules adopt unique three-dimensional structures that are required to perform their biological function under the normal life conditions. These three-dimensional structures are stabilized by relatively weak noncovalent intra- or intermolecular interactions that can be perturbed by changes in temperature, *T*, pressure, *p*, and the composition of the surrounding medium. The application of high pressures is of general physicochemical and biological interest, e.g. for understanding the physiology of organisms living in the deep sea.^1−9^ An increase in pressure changes the stability of protein and nucleic acid structures by shifting their population toward structures that occupy smaller volumes (Le Chatelier’s principle).^10−17^ The volumetric properties of biomacromolecules thus determine the effect of pressure on the equilibrium between the folded (F) and unfolded (U) states. For many biomolecules this equilibrium can be described by a two-state mechanism
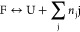
1which assumes that species j (e.g., ions, water molecules, cosolutes, cosolvents) exchange upon unfolding. Their apparent number, *n*_j_, may be positive (release of j) or negative (uptake of j). An increase in pressure shifts the equilibrium (eq 1) and consequently the thermal stability of the biomacromolecule (“melting” temperature), *T*_m_, which is defined as the temperature at which the populations of F and U species are equal. At a constant concentration of species j, the pressure dependence of *T*_*m*_ can be described by the Clapeyron equation
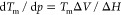
2where Δ*V* and Δ*H* represent the volume and enthalpy of unfolding, both of which depend on *T* and *p*. Therefore, d*T*_m_/d*p* is expected to vary with *T* and *p*, meaning that in general *T*_m_ versus *p* (pseudo)phase transition curves are not linear.^18−21^ However, for many different DNA structures a linear dependence of *T*_m_ on *p* is systematically observed in a broad range of pressures from 0.1 up to 200 MPa.^22−32^ In other words, why the slope, d*T*_m_/d*p*, is independent of *p* in this wide pressure range is not understood.

Here we examine the underlying thermodynamic reasons for this behavior. We demonstrate that the linear dependence of *T*_m_ on pressure (*p*) primarily arises from the specific relationship between compressibility and expansibility, both of which largely depend on DNA hydration.^33,34^ The presented relationship allows estimation of the compressibility of DNA unfolding using Δ*V*, Δ*H* and the corresponding expansibility, and suggests how compressibility and expansibility of unfolding depend on *T* and *p*. We also predict the existence of a convergence temperature at which Δ*V*, d*T*_m_/d*p*, and compressibility of unfolding simultaneously become equal to zero.

First, our analysis will provide thermodynamic reasons why the slope, d*T*_m_/d*p*, is independent of *p* at any selected solution composition. Hereafter we refer to the slope as *s* = *dT*_m_/*dp*. Pressure independence of *s* means that at constant solution composition:
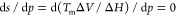
3

Taking the derivative of eq 3 it follows that
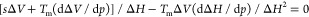
4

Under the studied conditions the enthalpy of unfolding, Δ*H*, is always a positive quantity. Multiplication with Δ*H* leads to the expression:
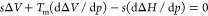
5

The dependence of Δ*V* on *T* and *p* can be expressed as
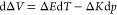
6where Δ*E* = (∂Δ*V*/∂*T*)_*p*_ represents the expansibility of unfolding while Δ*K* = −(∂Δ*V*/∂*p*)_*T*_ is the corresponding isothermal compressibility. At *T* = *T*_m_ dividing eq 6 by d*p* gives
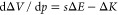
7

The dependence of Δ*H* on *T* and *p* is given as
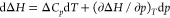
8where  is the heat capacity of unfolding. Moreover, the thermodynamic equation of state defines (∂Δ*H*/∂*p*)_*T*_ as
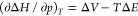
9

Inserting eq 9 into eq 8 and dividing so obtained eq 8 by d*p* defines the pressure dependence of Δ*H* on the *T*_m_ versus *p* line as
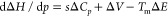
10

The combination of eqs 5, 7 and 10 results in the relation
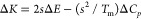
11

Equation 11 shows that the observed linear dependence of *T*_m_ on *p* postulates the relationship between Δ*K*, Δ*E* and Δ*C*_*p*_. Is the relation between Δ*K*, Δ*E* and Δ*C*_*p*_ surprising? No, since for DNA unfolding all three quantities are closely related to changes in hydration. For duplexes and quadruplexes around room temperature Δ*K* < 0, Δ*E* > 0 and Δ*C*_*p*_ > 0.^27,33^ The observed decrease in compressibility and increase in expansibility and heat capacity upon unfolding are in mutual agreement and suggest that the number of water molecules solvating the unfolded state is higher than that solvating the folded state, which is consistent with larger solvent accessible surface area of the unfolded state.^33^ For aqueous solutions, the isothermal compressibility, Δ*K*, is equal within experimental error to the corresponding adiabatic compressibility, Δ*K*_*S*_, which can be obtained from ultrasound velocity measurements.^33^ Experimental values for all these quantities (Δ*K* = Δ*K*_*S*_, Δ*E*, Δ*C*_*p*_, *s*) are known only for some polynucleotide duplex and G-quadruplex DNA.^22−27,34−40^ These data (presented in Table S1) suggest that in eq 11 the first term, 2*s*Δ*E*, is the one that predominantly determines Δ*K*, while the second term, (*s*^2^/*T*_m_)Δ*C*_*p*_, is significantly smaller in magnitude. By neglecting the second term the eq 11 simplifies into
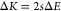
12

In the following, we show that this equation enables a quantitative analysis of the dependence of Δ*K* and Δ*E* on temperature and pressure. Since the slope, *s*, is constant on the *T*_m_ versus *p* line, eq 12 suggests that Δ*E* and Δ*K* must have a specific dependence on pressure and temperature, which is investigated next. Ideal species behavior can be assumed for the dilute solutions used in (un)folding studies. Therefore, the partial molar quantities  and the corresponding quantities of unfolding  can be considered composition-independent. If *T*_m_ is varied by changing the composition of the solution at a given *p* (changing the concentration of species j, e.g. the concentration of sodium ions), then the corresponding Δ*F* change is only due to its temperature dependence and not due to the changed solution composition. This property can be used to evaluate the temperature dependence of Δ*E*. It has been observed that at *p* = const. the dependence of Δ*V* on *T*_m_ is linear,^22−31^ which means that its slope, Δ*E*, can be considered independent of *T* and the solution composition. Based on this the dependence of Δ*K* on *T* can be analyzed by the temperature derivative of eq 12:
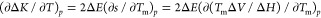
13

Given that heat capacity of the unfolding Δ*C*_*p*_ is a positive quantity, Δ*H* increases with increasing *T*_m_. Thus, when the numerator *T*_m_ in the *T*_m_/Δ*H* ratio increases, the denominator also increases to an extent that in the studied temperature interval the *T*_m_/Δ*H* ratio can be considered constant within the experimental error. Under these assumptions, the derivative  in eq 13 can be expressed as
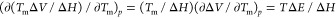
14while (∂Δ*K*/∂*T*)_*p*_ as
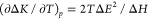
15Since *T*/Δ*H* and Δ*E* can be regarded as temperature-independent (see above), the derivative (∂Δ*K*/∂*T*)_*p*_ can also be taken as a temperature-independent quantity. Thus, the integration of eq 15 (for *p* = *p*_0_) results in the relation
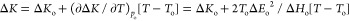
16where Δ*H*_0_ = Δ*H*(*T*_0_,*p*_0_), Δ*E*_0_ = Δ*E*(*p*_0_) and Δ*K*_0_ = Δ*K*(*T*_0_,*p*_0_) are the quantities defined at the selected reference state (*T*_0_,*p*_0_). By expressing Δ*K*_0_ form eq 12, we obtain
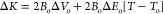
17where *B*_0_ = *T*_0_Δ*E*_0_/Δ*H*_0_. Equation 17 thus predicts that the dependence of Δ*K* on *T* is linear, in agreement with the experimental observations.^34,38^ More importantly, eq 17 enables comparison between the calculated Δ*K*(*T*,*p*_0_) values with the corresponding experimental Δ*K*_*S*_(*T*,*p*_0_) values. In other words, Δ*K*(*T*,*p*_0_) can in principle be estimated from the corresponding Δ*V*, Δ*E*and Δ*H* values. To compare the validity of the estimated values at the same temperature, the experimental Δ*K*_*S*_ measured at temperature *T*_S_ (Table S1) were extrapolated to the selected *T* using eq 16: Δ*K*_S_ = Δ*K*_S_(*T*_S_) + (∂Δ*K*/∂*T*)_*p*_0__(*T* – *T*_S_). We observe a good agreement between experimental Δ*K*_S_ and Δ*K* calculated using eq 17 (Figure 1), with the slope of the correlation line around one, which justifies the approximations applied in deriving eq 17. The largest deviation from the correlation line is observed for poly[d(A)]poly[d(T)] (Figure 1). For G-quadruplexes the predicted Δ*K* [given per mole of bases, 25 °C, 1 bar values between −6 × 10^4^ and −15 × 10^4^ mL mol_b_^–1^ bar^–1^] are significantly different from those of the polynucleotide duplexes [values between 0 and −10 × 10^4^ mL mol_b_^–1^ bar^–1^]. On the other hand, the slope (∂Δ*K*/∂*T*)_*p*_0__ =  appears to be positive and similar for the two sets of different DNA structures analyzed here [average value: (0.14 ± 0.06) × 10^4^ mL mol_b_^–1^ bar^–1^ K^–1^], which is in agreement with the previous observation that the temperature dependence of Δ*K* is relatively insensitive to the type of the studied DNA.^34^ Taken together, these findings suggest that the temperature dependence of Δ*K* can be successfully estimated using eq 17.

**Figure 1:**
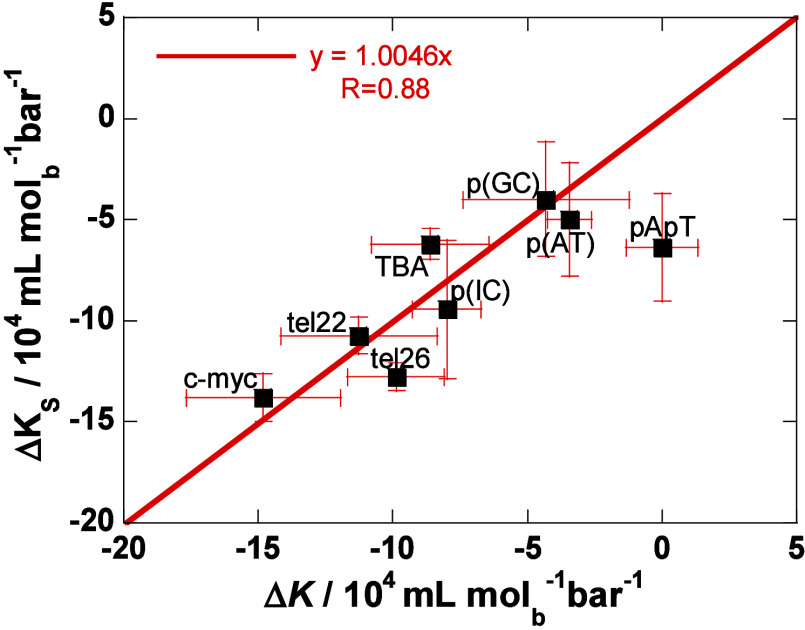
Correlation between the measured Δ*K*_S_ and calculated Δ*K* (eq 17, *p*_0_ = 1 bar, *T* = 25 °C) for polynucleotide duplexes and G-quadruplexes (Table S1). Values are given per mol of base.

The next question is how Δ*E* depends on pressure and how this affects the temperature dependence of Δ*K*. Experimental observations suggest that Δ*E* can be considered as a positive, temperature-independent quantity at constant pressure. From Maxwell’s relation it follows that (∂Δ*E*/∂*p*)_*T*_ = −(∂Δ*K*/∂*T*)_*p*_ = −2*T*Δ*E*^2^/Δ*H*. Accordingly, the dependence of Δ*E* on *p* at given *T* can be estimated by integration of the differential equation
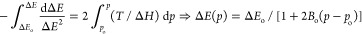
18where *T*/Δ*H* ratio was assumed to be pressure-independent (*T*/Δ*H* = *T*_0_/Δ*H*_0_). Figure 2 shows that Δ*E* decreases nonlinearly with increasing pressure. It should be noted that the inclusion of meaningful experimentally based estimates of the *T*/Δ*H* pressure dependence does not significantly change the predicted pressure dependence of Δ*E*.

**Figure 2:**
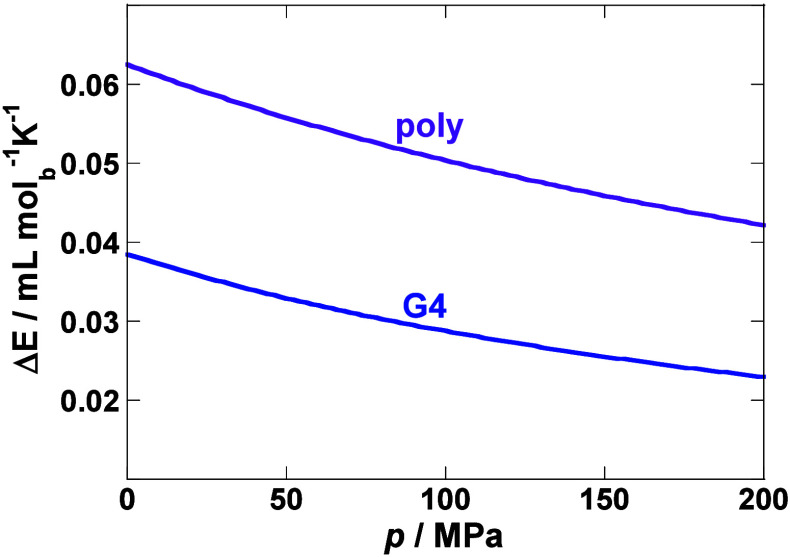
Δ*E* estimated as a function of pressure by eq 18. Estimation is based on average values for here analyzed polynucleotide duplexes (poly) and G-quadruplexes (G4) (Table S2, reference state: *p*_0_ = 1 bar, *T*_0_ = 25 °C). Values are given per mol of base.

Maxwell’s relation (∂Δ*E*/∂*p*)_*T*_ = −(∂Δ*K*/∂*T*)_*p*_ shows how the pressure dependence of Δ*E* affects the temperature dependence of Δ*K*. Using eq 12, Δ*K*(*p*) = 2*s*(*p*)Δ*E*(*p*), we will now analyze how pressure affects Δ*K* at constant temperature. Since we consider Δ*K* to be independent of the composition, the problem can be translated into analyzing Δ*K* as a function *p* at constant *T*_m_. The fact is that a change in *p* is accompanied by a change in *T*_m_ at constant solution composition. Therefore, a change in *p* at constant *T*_m_ is only possible when the change in *T*_m_ due to a change in pressure exactly compensates the change in *T*_m_ due to change in composition. In this way, the equation for *s*(*T*_m_, *p*) was derived (see SI)
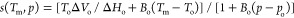
19

The combination of eqs 12, 18, and 19 results in
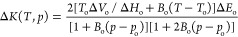
20where *B*_0_ = *T*_0_Δ*E*_0_/Δ*H*_0_. Equation 20 describes Δ*K* as a function of the independent variables *T* and *p*, which is for polynucleotide duplexes and G-quadruplexes presented in Figure 3.

**Figure 3:**
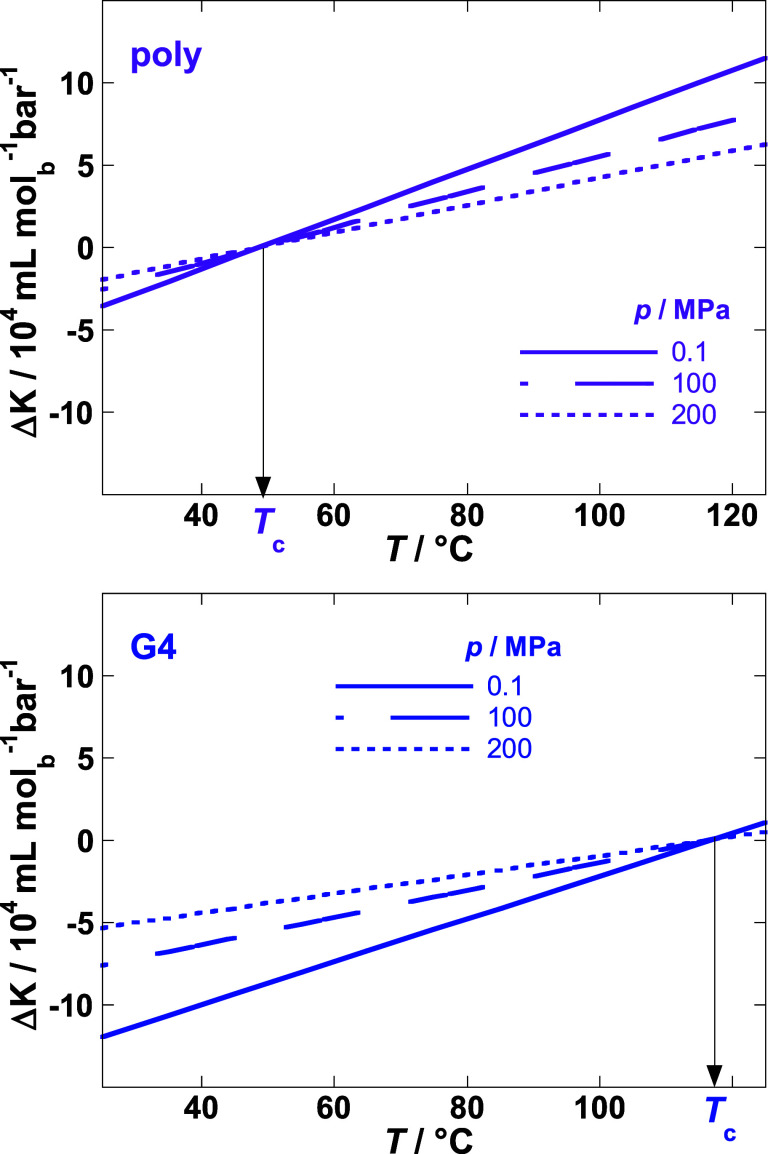
Δ*K* estimated as a function of temperature at different pressures by eq 20. Estimations are based on average values for here analyzed polynucleotide duplexes (poly) and G-quadruplexes (G4) (Table S2, reference state: *p*_0_ = 1 bar, *T*_0_ = 25 °C). Values are given per mol of base. *T*_c_ represents a convergence temperature at which Δ*K*, Δ*V* and *s= dT*_m_/*dp* become equal to zero.

Finally, we show that the Δ*K* versus *T* and *p* data (Figure 3) contain all the necessary information on the thermal stability of DNA as a function of pressure. Namely, the ratio of the derivatives −(∂Δ*K*/∂*p*)_*T*_/(∂Δ*K*/∂*T*)_*p*_, which can be estimated from these data, is directly related to d*T*_m_/d*p* (see derivation in SI)
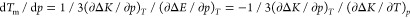
21

Figure 3 shows that (∂Δ*K*/∂*T*)_*p*_ is positive and decreases with increasing *p*. This is because Δ*E* decreases as *p* increases [(∂Δ*K*/∂*T*)_*p*_ = 2*T*Δ*E*^2^/Δ*H*, Figure 2]. On the other hand, (∂Δ*K*/∂*p*)_*T*_ changes with *T* from positive values at *T* < *T*_c_ (⇒ d*T*_m_/d*p* < 0) to negative values at *T* > *T*_c_ (⇒ d*T*_m_/d*p* > 0), where *T*_c_ represents the convergence temperature at which Δ*K* reaches exactly zero. It follows from eq 20 that the convergence temperature *T*_c_ is independent of pressure and can be estimated as
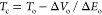
22

Since Δ*E* > 0, it follows from eq 12 that Δ*K* = 0 when Δ*V* = 0 (resulting in *s* = 0). Thus, the observed linear dependence of *T*_m_ on *p* through the derived eq 12 postulates the existence of the convergence temperature at which Δ*K*, Δ*V* and *s* = d*T*_m_/d*p* simultaneously become equal to zero. The estimated *T*_c_ values for polynucleotide duplexes range between 45 to 65 °C (with the exception of poly[d(A)]poly[d(T)]), while for quadruplexes they range between 100 and 145 °C, which is in line with the reported temperature dependencies of Δ*V* and Δ*K*.^27,34,38^ Obviously, the mutual compensation of the contributions within Δ*V* and Δ*K* and their specific temperature dependencies cause Δ*V* and Δ*K* to change with temperature from negative (at *T < T*_c_) to positive (at *T > T*_c_) values. The prediction that this occurs at the same temperature (*T*_c_) for both Δ*V* and Δ*K* suggests that hydration contributions play the most important role in this compensation.^29^ What causes the sign change of Δ*V* for DNA duplex unfolding at around 50 °C has been explained by Makhatadze, Marky and co-workers in the recent article.^41^ Following their analysis, Δ*V* can be considered as the sum of two contributions: Δ*V*= Δ*V*_*hyd*_ + Δ*V*_*other*_. Δ*V*_*hyd*_ represents the volume change associated with the interactions of the folded and unfolded states with water (i.e., hydration volume). Δ*V*_*other*_ represents the contributions associated with the conformational changes of DNA (i.e., intrinsic volume change of the DNA + thermal volume change indicating the change of the void space). Δ*V*_*other*_ appears to be temperature independent and has a negative sign. However, the Δ*V*_*hyd*_ contribution is positive and increases with increasing temperature since expansibility Δ*E*, which is determined predominately by the hydration contribution,^33^ is a positive quantity. At *T < T*_c_ (∼50 °C) it appears that the positive values of Δ*V*_*hyd*_ are not sufficient to overcome the negative values of Δ*V*_*other*_, so that Δ*V* < 0. Increasing the temperature increases Δ*V*_*hyd*_ that overcomes the negative Δ*V*_*other*_ at *T > T*_c_. As a result, the value of Δ*V* becomes positive at temperatures above 50 °C. Why are the convergence temperatures for Δ*V* and Δ*K* very similar (equal within our approximations)? This is a direct consequence of the observed linearity which postulates that at a given pressure Δ*K* is proportional to Δ*V* (eq 12). Δ*K* itself is dominated by the hydration contribution^33^ that is negative at *T < T*_c_ and increases with temperature resulting in positive values of Δ*K* at *T > T*_c_.

Why is *T*_c_ for G-quadruplexes very different (>50 °C higher) than for duplexes? In contrast to duplex, Δ*V*_*other*_ for G-quadruplex unfolding has a positive sign.^27,35^ Its magnitude is significantly lower than that of Δ*V*_*hyd*_ that has a negative sign,^27,35^ resulting in Δ*V* < 0 at lower temperatures. However, Δ*V*_*hyd*_ becomes less negative at higher temperatures since expansibility Δ*E*, which is for G-quadruplexes also determined predominately by the hydration contribution,^27^ is a positive quantity. It appears that the reason that *T*_c_ (G-quadruplex) ≫ *T*_c_ (duplex) is 2-fold. At low temperature the compensation of the contributions results in Δ*V* that is significantly more negative for G-quadruplexes than for duplexes (Table S2). In addition, the expansibility that determines the temperature dependence of Δ*V* (and Δ*K* - eq 12) is significantly lower for G-quadruplexes (Table S2) meaning that increasing of Δ*V* and Δ*K* with temperature is less intense for G-quadruplexes than for duplexes. Interestingly, *T*_c_ for G-quadruplexes is very similar to the convergence temperature at which the entropy of transfer of hydrophobic groups of globular proteins from their core to water reaches zero.^42^ In other words, the volumetric properties of the unfolding of G-quadruplexes, which have a globular structure stabilized by dehydration, are more similar to those of globular proteins than to those of DNA duplexes.^43^

Taken together, our analysis suggests that the pressure and temperature dependencies of Δ*K* and Δ*E* need to be considered when extrapolating the thermodynamic parameters of DNA unfolding to different *p* and *T*. Figure 4 shows that they must be taken into account when extrapolating Δ*V* (see eq S16). In contrast, when extrapolating the enthalpy, Δ*H*, the entropy, Δ*S*, and the Gibbs free energy, Δ*G*, the consideration of the *p* and *T* dependence of Δ*K* and Δ*E* does not play a major role (Figure S2, eqs S17–S21). The estimates of Δ*H*, Δ*S*, and Δ*G* at pressures up to ∼200 MPa appear to be reliable within the experimental error, even if we consider Δ*K* and Δ*E* as *p-* and *T*-independent quantities.

**Figure 4:**
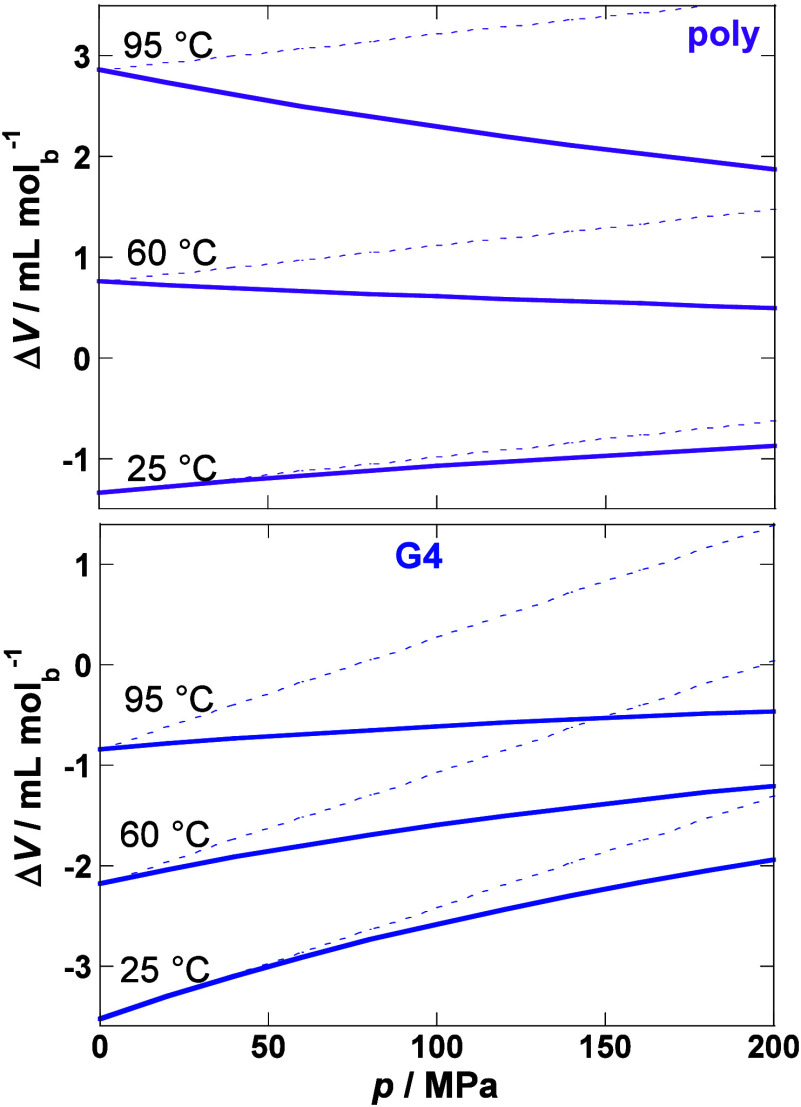
Δ*V* estimated as a function of pressure at different temperatures by eq S16 (Full lines – *p* and *T* dependencies of Δ*K* and Δ*E* are considered; dotted lines - Δ*K* and Δ*E* are taken as fixed values from Table S2). Estimations are based on average values for here analyzed polynucleotide duplexes (poly) and G-quadruplexes (G4) (Table S2, reference state: *p*_0_ = 1 bar, *T*_0_ = 25 °C). Δ*V* is given per mol of base.

In conclusion, the presented analysis shows that the compressibility Δ*K* and the expansibility Δ*E* are the key variables that determine the pressure dependence of the thermal stability of DNA. Using the thermodynamic equation of state and Maxwell’s relation, we predicted Δ*K* and Δ*E* as functions of temperature and pressure. These predictions suggest the existence of a convergence temperature, *T*_c_, at which Δ*V*, *dT*_m_/*dp*, and Δ*K* simultaneously become equal to zero. While the observed volumetric and stability parameters differ between the duplexes and G-quadruplexes studied here, the slope (∂Δ*K*/∂*T*)_*p*_ is very similar, likely due to a similar effect of temperature on nonspecific DNA hydration. We emphasized the importance of the hydration term as the dominant factor in defining the sign of Δ*V* and Δ*K*. At *T < T*_c_ the folded DNA structure is pressure unstable, since Δ*V* is negative (i.e., an increase in pressure leads to unfolding). At *T > T*_c_ Δ*V* becomes positive and the DNA structure is stabilized by an increase in pressure. Furthermore, our analysis suggests that *T*_c_ is pressure-independent. In this light, pressure induced destabilization and stabilization effects depend only on temperature and can be considered independent of the initial pressure at which the pressure perturbation is applied.

Overall, the thermodynamic analysis provides interesting new relationships between thermodynamic quantities that determine the pressure- and temperature-dependent changes in DNA stability.

## Supporting Information Available

Supplementary tables, figures and equations (PDF)
Transparent Peer Review report available (PDF)
