# Supplementary material for: Thermodynamic Origin of the Linear Pressure Dependence of DNA Thermal Stability
Source: J Phys Chem Lett. 2024 Aug 28;15(35):9064–9. doi: 10.1021/acs.jpclett.4c01563 (PMC11382263; doi:10.1021/acs.jpclett.4c01563)
Supplement: Supplementary file 1 — jz4c01563_si_001.pdf [file jz4c01563_si_001.pdf]

Thermodynamic Origin of the Linear Pressure Dependence of DNA Thermal Stability

Jurij Lah, San Hadži**

Faculty of Chemistry and Chemical technology, University of Ljubljana, Večna pot 113, 1000 Ljubljana, Slovenia

AUTHOR INFORMATION

Corresponding Authors

* Jurij Lah, e-mail: jurij.lah@fkkt.uni-lj.si

* San Hadži, e-mail: san.hadzi@fkkt.uni-lj.si

SUPPORTING INFORMATION (SI)

Table S1: Thermodynamic parameters of DNA unfolding (p_o = 1 bar)

DNA	T_o / °C	ΔH_o / kJ mol^{-1}	ΔC_p / kJ mol^{-1} K^{-1}	ΔV_o / mL mol^{-1}	ΔE_o / mL mol^{-1} K^{-1}	T_S / °C	ΔK_S / 10^4 mL mol^{-1} bar^{-1} K^{-1}
poly[d(AT)][1-3]	35.8 ± 0.2	14.5 ± 0.5	0.21 ± 0.07	-0.70 ± 0.11	0.06 ± 0.01	51.0	-1 ± 2
poly[d(A)]poly[d(T)][2-4]	59.2 ± 0.2	18.2 ± 0.5	0.27 ± 0.03	1.72 ± 0.12	0.05 ± 0.01	56.5	-3.5 ± 2
poly[d(GC)][2,3,5]	106.5 ± 0.3	20.2 ± 0.5	0.27 ± 0.03	2.40 ± 0.28	0.05 ± 0.01	60.0	0 ± 2
poly[d(IC)][1-3]	29.0 ± 0.2	16.2 ± 0.5	0.29 ± 0.06	-2.18 ± 0.16	0.08 ± 0.01	60.0	0 ± 2
tel22 (22 bases)[6,7]	40.0 ± 0.5	174 ± 3	1.6 ± 0.1	-66.0 ± 2.0	0.87 ± 0.16	25.0	-236 ± 20
tel26 (26 bases)[6-8]	60.6 ± 0.2	170 ± 3	1.7 ± 0.1	-38.3 ± 3.7	0.92 ± 0.07	25.0	-332 ± 18
c-myc (22 bases)[7,9]	83.4 ± 1.1	129 ± 3	1.7 ± 0.1	-16.9 ± 1.8	0.87 ± 0.07	25.0	-304 ± 26
TBA (15 bases)[7,10,11]	52.6 ± 1.4	211 ± 9	1.1 ± 0.1	-54.6 ± 4.2	0.59 ± 0.07	20.0	-98 ± 10

- ΔK_S values for poly[d(GC)] and poly[d(IC)] were estimated from the ΔK_S versus T_m correlation line presented in ref 3.
- ΔK_S value for TBA was obtained from ref 11.
- ΔV was extrapolated to T_o using ΔE_o.
- In the absence of reliable measured ΔE_o and ΔC_p values for c-myc and TBA, they were assumed to be proportional to the no. of bases. Their values were estimated as (no. of bases) x (value for tel22) / 22. ΔC_p values for tel22 were obtained from ref 7.

Table S2: Average thermodynamic parameters of DNA unfolding (p_o = 1 bar) extrapolated to T_o = 25 °C using the data presented in Table S1.

	polymeric duplex	G-quadruplex
ΔH_o / kJ mol$_b^{-1}$	13 ± 2	6 ± 4
ΔS_o / kJ mol$_b^{-1}$ K^{-1}	0.04 ± 0.01	0.02 ± 0.01
ΔC_p / kJ mol$_b^{-1}$ K^{-1}	0.13 ± 0.01	0.07 ± 0.01
ΔV_o / mL mol$_b^{-1}$	-1.3 ± 1.0	-3.5 ± 0.8
ΔE_o / mL mol$_b^{-1}$ K^{-1}	0.06 ± 0.02	0.04 ± 0.01
ΔK_o / 10^4 mL mol$_b^{-1}$ bar^{-1} K^{-1}	-4 ± 3	-11 ± 2
$\Delta K_{S,o}$ / 10^4 mL mol$_b^{-1}$ bar^{-1} K^{-1}	-6 ± 2	-11 ± 3
$(\partial \Delta K/\partial T)_{p_o}$/ 10^4 mL mol$_b^{-1}$ bar^{-1} K^{-2}	0.15 ± 0.08	0.13 ± 0.04

Parameters are given per mol of base.

Derivation of Eq. 19 (main text): $\boldsymbol{s(T_m, p) = [T_o \Delta V_o/\Delta H_o + B_o(T_m - T_o)]/[1 + B_o(p - p_o)]}$

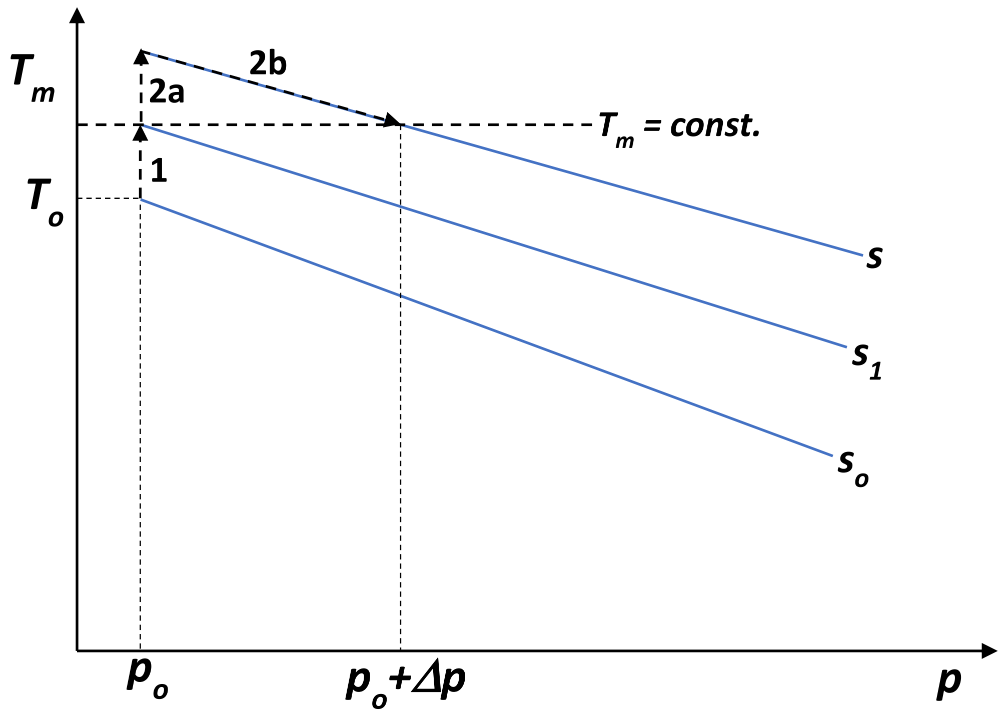

Figure S1. Graphic presentation.

1) **Temperature dependence of *s* at *p* = const.** Changing composition at $p = p_o$ is accompanied by change in T_m ($T_o \rightarrow T_m$) and according to Eq. 14 (main text) to the corresponding change in s (see Fig. S1):

$$s_1 - s_o = B_o(T_m - T_o); \; B_o = T_o \Delta E_o/\Delta H_o \qquad \text{(S1)}$$

2) **Pressure dependence of *s* at T_m = const.** Changing p and keeping T_m constant is possible with the corresponding change in the composition in a way that change in T_m due to varying composition (**2a**) (see Fig. S1):

$$\Delta T_m(2a) = (s - s_1)/B_o \qquad \text{(S2)}$$

and the change in T_m due to varying pressure (**2b**):

$$\Delta T_m(2b) = s\Delta p \qquad \text{(S3)}$$

are exactly compensated:

$\Delta T_{\mathrm{m}}(2\mathrm{a}) + \Delta T_{\mathrm{m}}(2\mathrm{b}) = 0$ (S4)

Combination of Eqs S1-S4 gives

$(s - s_1)/B_{\mathrm{o}} = -s\Delta p \ \Rightarrow\ s = s_1/(1 + B_{\mathrm{o}}\Delta p) \ \Rightarrow\ s = (s_{\mathrm{o}} + B_{\mathrm{o}}(T_{\mathrm{m}} - T_{\mathrm{o}}))/(1 + B_{\mathrm{o}}\Delta p) \ \Rightarrow$

$\boldsymbol{s(T_{\mathrm{m}}, p) = [T_{\mathrm{o}}\Delta V_{\mathrm{o}}/\Delta H_{\mathrm{o}} + B_{\mathrm{o}}(T_{\mathrm{m}} - T_{\mathrm{o}})]/[1 + B_{\mathrm{o}}(p - p_{\mathrm{o}})]}$ (S5)

Derivation of Eq. 22 (main text): $\boldsymbol{s = dT_{\mathrm{m}}/dp = -1/3(\partial\Delta K/\partial p)_{T_{\mathrm{m}}}/(\partial\Delta K/\partial T_{\mathrm{m}})_p}$

$\Delta K = 2\mathrm{s}\Delta E$ (S6)

$d\Delta K/dp = 2s(d\Delta E/dp)$ (S7)

$\frac{d\Delta K}{dp} = \left(\frac{\partial\Delta K}{\partial p}\right)_{T_{\mathrm{m}}}\left(\frac{dp}{dp}\right) + \left(\frac{\partial\Delta K}{\partial T_{\mathrm{m}}}\right)_p\left(\frac{dT_{\mathrm{m}}}{dp}\right)$ (S8)

$\frac{d\Delta K}{dp} = \left(\frac{\partial\Delta K}{\partial p}\right)_{T_{\mathrm{m}}} + \left(\frac{\partial\Delta K}{\partial T_{\mathrm{m}}}\right)_p s$ (S9)

$\frac{d\Delta E}{dp} = \left(\frac{\partial\Delta E}{\partial p}\right)_{T_{\mathrm{m}}}\left(\frac{dp}{dp}\right) + \left(\frac{\partial\Delta E}{\partial T_{\mathrm{m}}}\right)_p\left(\frac{dT_{\mathrm{m}}}{dp}\right)$ (S10)

$\left(\frac{\partial\Delta E}{\partial T_{\mathrm{m}}}\right)_p = 0$ (S11)

$\frac{d\Delta E}{dp} = \left(\frac{\partial\Delta E}{\partial p}\right)_{T_{\mathrm{m}}} = -\left(\frac{\partial\Delta K}{\partial T_{\mathrm{m}}}\right)_p$ Maxwell's relation (S12)

Combination of S7, S9 and S12 gives

$\left(\frac{\partial\Delta K}{\partial p}\right)_{T_{\mathrm{m}}} + \left(\frac{\partial\Delta K}{\partial T_{\mathrm{m}}}\right)_p s = -2s\left(\frac{\partial\Delta K}{\partial T_{\mathrm{m}}}\right)_p$ (S13)

and finally

$\boldsymbol{s = dT_{\mathrm{m}}/dp = -1/3(\partial\Delta K/\partial p)_{T_{\mathrm{m}}}/(\partial\Delta K/\partial T_{\mathrm{m}})_p}$ **(S14)**

Extrapolation of thermodynamic state function differences ($\Delta F = \Delta V$, ΔH, ΔS, ΔG) from the reference state (T_o, p_o) to the state at any selected temperature (T) and pressure (p) at constant composition is based on the integration of the corresponding total differentials ($\mathrm{d}\Delta F$). Since the obtained overall differences $\Delta F(T,p)$ - $\Delta F_o(T_o, p_o)$ are independent of path, the integration was performed in two steps:

1. temperature was changed from T_o to T at p_o = const.
2. pressure was changed from p_o to p at T = const.

In equations below $B_o = T_o\Delta E_o/\Delta H_o$ and $f(p) = B_o(p - p_o)$

Volume change:

$\mathrm{d}\Delta V = \Delta E\mathrm{d}T - \Delta K\mathrm{d}p$ (S15)

$\Delta V = \Delta V_o + \Delta E_o(T - T_o) - \int_{p_o}^{p} \Delta K\,\mathrm{d}p$ (S16)

a) ΔK is considered independent of T and p: $\int_{p_o}^{p} \Delta K\,\mathrm{d}p = \Delta K_o(p - p_o)$

b) $\Delta K = \frac{2[T_o\Delta V_o/\Delta H_o + B_o(T - T_o)]\Delta E_o}{[1+f(p)][1+2f(p)]}$:
$\int_{p_o}^{p} \Delta K\,\mathrm{d}p = 2(\Delta V_o + \Delta E_o(T - T_o)) \cdot \ln\,[(1 + 2f(p))/(1 + f(p))]$

Enthalpy change:

$\mathrm{d}\Delta H = \Delta C_p\mathrm{d}T + (\Delta V - T\Delta E)\mathrm{d}p$ (S17)

$\Delta H = \Delta H_o + \Delta C_p(T - T_o) + [\Delta V_o + \Delta E_o(T - T_o)](p - p_o) - \int_{p_o}^{p}(\int_{p_o}^{p} \Delta K\,\mathrm{d}p)\mathrm{d}p - T\int_{p_o}^{p} \Delta E\mathrm{d}p$ (S18)

a) ΔK is considered independent of T and p: $\int_{p_o}^{p}(\int_{p_o}^{p} \Delta K\,\mathrm{d}p)\mathrm{d}p = 0.5\Delta K_o(p - p_o)^2$
ΔE is considered independent of T and p: $\int_{p_o}^{p} \Delta E\mathrm{d}p = \Delta E_o(p - p_o)$

b) $\Delta K = \frac{2[T_o\Delta V_o/\Delta H_o + B_o(T - T_o)]\Delta E_o}{[1+f(p)][1+2f(p)]}$, $\Delta E = \Delta E_o/[1 + 2f(p)]$
$I_1 = ((1+f(p))\cdot(\ln(1+f(p))-1)+1)/B_o$
$I_2 = ((1+2f(p))\cdot(\ln(1+2f(p))-1)+1)/(2B_o)$
$\int_{p_o}^{p}(\int_{p_o}^{p} \Delta K\,\mathrm{d}p)\mathrm{d}p = 2[\Delta V_o + \Delta E_o(T - T_o)] \cdot (I_2 - I_1)$
$\int_{p_o}^{p} \Delta E\mathrm{d}p = (\Delta E_o/(2B_o)) \cdot ln(1 + 2f(p))$

Entropy change:

$\mathrm{d}\Delta S = (\Delta C_p/T)\mathrm{d}p - \Delta E\mathrm{d}p$ (S19)

$\Delta S = \Delta S_o + \Delta C_p ln(T/T_o) - \int_{p_o}^{p} \Delta E\mathrm{d}p$ (S20)

a) ΔE is considered independent of T and p: $\int_{p_o}^{p} \Delta E\mathrm{d}p = \Delta E_o(p - p_o)$

b) $\Delta E = \Delta E_o/[1 + 2f(p)]$: $\int_{p_o}^{p} \Delta E\mathrm{d}p = (\Delta E_o/(2B_o)) \cdot ln(1 + 2f(p))$

Gibbs free energy change (at given T and p):

$\Delta G = \Delta H - T\Delta S$ (S21)

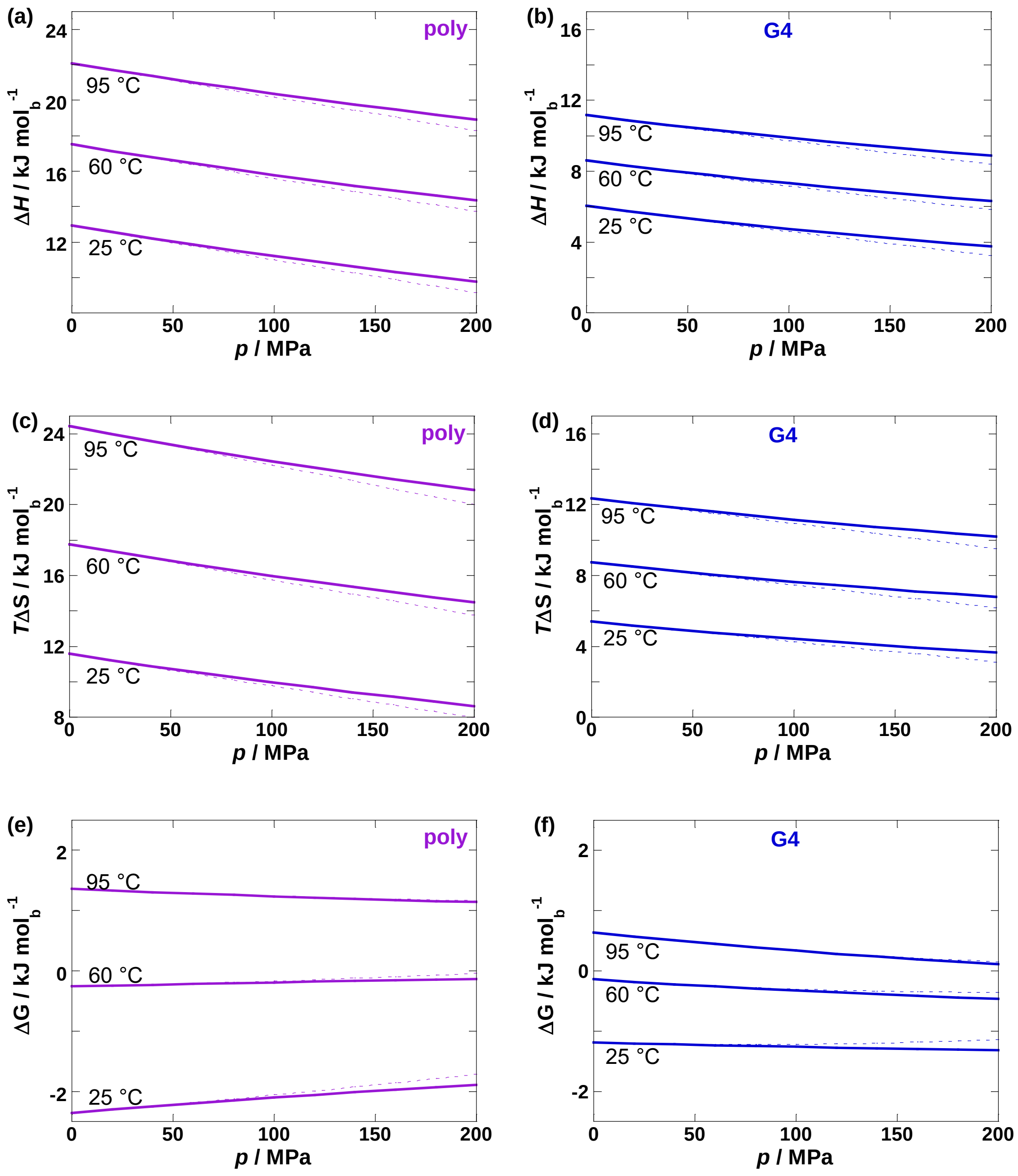

Figure S2. ΔH, $T\Delta S$ and ΔG estimated as a function of pressure at different temperatures by Eqs. S15-S21. (Full lines – p and T dependencies ΔK and ΔE are considered; dotted lines - ΔK and ΔE are taken as fixed values from Table S2). Estimations are based on average values for here analyzed polymeric duplexes (poly) and G-quadruplexes (G4) (Table S2, reference state: p_o = 1 bar, T_o = 25 °C). ΔH, $T\Delta S$ and ΔG are given per mol of base.

REFERENCES

(1) Rayan, G.; Macgregor, R. B. Jr. Comparison of the Heat- and Pressure-Induced Helix-Coil Transition of Two DNA Copolymers. *J. Phys. Chem. B* **2005**, *109*, 15558–15565, DOI: 10.1021/jp050899c

(2) Chalikian, T. V.; Breslauer, K. J. Volumetric Properties of Nucleic Acids. *Biopolymers* **1998**, *48*, 264–280, DOI: 10.1002/(SICI)1097-0282(1998)48:4<264::AID-BIP6>3.0.CO;2-8

(3) Chalikian, T. V., Völker, J. G.; Plum, G. E.; Breslauer, K. J. A More Unified Picture for the Thermodynamics of Nucleic Acid Duplex Melting: A Characterization by Calorimetric and Volumetric Techniques. *Proc. Natl. Acad. Sci. U.S.A.* **1999**, *96*, 7853–7858, DOI: 10.1073/pnas.96.14.7853

(4) Wu, J. Q.; Macgregor, R. B. Jr. Pressure Dependence of the Melting Temperature of dA.dT Polymers. *Biochemistry* **1993**, *32*, 12531–12537, DOI: 10.1021/bi00097a033

(5) Wu, J. Q.; Macgregor, R. B. Jr. Pressure Dependence of the Helix-Coil Transition Temperature of Poly [d(G-C)]. *Biopolymer*s **1995**, *35*, 369–376, DOI: 10.1002/bip.360350404

(6) Fan, H.Y.; Shek, Y. L.; Amiri, A.; Dubins, D. N.; Heerklotz, H.; Macgregor, R. B. Jr.; Chalikian, T. V. Volumetric Characterization of Sodium-Induced G-Quadruplex Formation. *J. Am. Chem. Soc.* **2011**, *133*, 4518–4526, DOI: 10.1021/ja110495c

(7) Bončina, M.; Vesnaver, G.; Chaires, J. B.; Lah, J. Unraveling the Thermodynamics of the Folding and Interconversion of Human Telomere G-Quadruplexes. *Angew. Chem. Int. Ed. Engl.* **2016**, *55*, 10340–10344, DOI: 10.1002/anie.201605350

(8) Shek, Y. L.; Noudeh, G. D.; Nazari, M.; Heerklotz, H.; Abu-Ghazalah, R. M.; Dubins, D. N.; Chalikian, T. V. Folding Thermodynamics of the Hybrid-1 Type Intramolecular Human Telomeric G-Quadruplex. *Biopolymers* **2014**, 101, 216–227, DOI: 10.1002/bip.22317

(9) Chalikian, T. V.; Macgregor, R. B. Jr. Volumetric Properties of Four-Stranded DNA Structures. *Biology (Basel)* **2021**, *10*, 813, DOI: 10.3390/biology10080813

(10) Takahashi, S.; Sugimoto, N. Effect of Pressure on the Stability of G-Quadruplex DNA: Thermodynamics under Crowding Conditions. *Angew. Chem. Int. Ed. Engl.* **2013**, *52*, 13774–13778, DOI: 10.1002/anie.201307714

(11) Kankia, B. I.; Marky, L. A. Folding of the Thrombin Aptamer into a G-Quadruplex with Sr(2+): Stability, Heat, and Hydration. *J. Am. Chem. Soc.* **2001**, *123*, 10799–10804, DOI: 10.1021/ja010008o
