# Supplementary material for: Thermodynamic Origin of the Linear Pressure Dependence of DNA Thermal Stability
Source: J Phys Chem Lett. 2024 Aug 28;15(35):9064–9. doi: 10.1021/acs.jpclett.4c01563 (PMC11382263; doi:10.1021/acs.jpclett.4c01563)
Supplement: Supplementary file 2 — jz4c01563_si_002.pdf [file jz4c01563_si_002.pdf]

jz-2024-015639.R1

Name: Peer Review Information for "Thermodynamic Origin of the Linear Pressure Dependence of DNA Thermal Stability"

First Round of Reviewer Comments

Reviewer: 1

Comments to the Author
The authors investigated the reason why the pressure dependence of DNA melting temperatures shows linearity. They precisely studied the trend using thermodynamic parameters including compressibility and expansibility. They also found that compressibility and volume of (un)folding become zero at a certain temperature. Since the relationship between Tm and pressure was well known in a linear fashion, the origin of the trend is of interest. Therefore, this study illuminates the chemistry behind the pressure-temperature dependent properties of DNA structures. Although this study includes informative knowledge, several points must be considered before publication.

1) On page 5, the authors explained that duplexes and quadruplexes show $\Delta K<0$, $\Delta E>0$, and $\Delta Cp>0$. First, the references are needed. Second, some duplexes showed the positive slope of dTm/dp, which was opposite to G-quadruplexes and should be $\Delta K>0$.

2) The authors claimed hydration is the key to the trend of Tm vs pressure. In general, DNA duplexes uptake water molecules when they folds (e.g., J. Phys. Chem. B 1999, 103, 41, 8759–8767), whereas G-quadruplexes release water molecules (e.g., J. Am. Chem. Soc. 2010, 132, 48, 17105–17107). Are all cases valid in the authors' treatment?

3) On page 6, to show the validity of equation 12, it seems that the authors demonstrated using reported values as shown in the sentence "These data suggest…". The data should be shown in the Supporting information.

4) The authors' interpretation that the correlation of Tm and pressure is explained as the linear function with the slope derived from the ratio of ΔK and ΔE. Then, how about the physical meaning of the intercept of the linear function?

5) The stability of DNA highly depends on the cation binding. However, the authors only discuss hydration, and this study did not include any information about the contribution of the cation binding.

6) The identification of Tc is interesting. What does Tc mean in physical chemistry and biochemistry of DNA melting? In the G-quadruplex, Tc shows higher than the boiling point of water.

Reviewer: 2

Comments to the Author
The analysis presented in this manuscript might be interesting. However, it appears the authors may not be cognizant of a recent publication that establishes a direct correlation between hydration changes and the volumetric properties of dsDNA (Makhatadze GI, Chen CR, Khutsishvili I, Marky LA (2022) The Volume Changes of Unfolding of dsDNA. Biophysical Journal, 121(24):4892-4899. doi:

10.1016/j.bpj.2022.08.005.) This BJ paper also discusses the temperature dependence of the volume changes, and thus the pressure dependence of DNA stability. It would be beneficial for the authors to frame their analysis within the context of this BJ paper, demonstrating what unique perspectives they can offer on DNA stability. . I am open to assessing a revised version. Regrettably, in its current form, the manuscript falls short in a number of categories with the “novelty” aspect being the most critical.

Author's Response to Peer Review Comments:

Reviewers comments and our answers

Reviewer: 1

Recommendation: This paper may be publishable, but major revision is needed; I would like to be invited to review any future revision.

Comments:
The authors investigated the reason why the pressure dependence of DNA melting temperatures shows linearity. They precisely studied the trend using thermodynamic parameters including compressibility and expansibility. They also found that compressibility and volume of (un)folding become zero at a certain temperature. Since the relationship between Tm and pressure was well known in a linear fashion, the origin of the trend is of interest. Therefore, this study illuminates the chemistry behind the pressure-temperature dependent properties of DNA structures. Although this study includes informative knowledge, several points must be considered before publication.

Answer: We thank the reviewer for his/her constructive comments and remarks and we are open to further discussion if any additional points need to be clarified.

1) On page 5, the authors explained that duplexes and quadruplexes show $\Delta K<0$, $\Delta E>0$, and $\Delta Cp>0$. First, the references are needed. Second, some duplexes showed the positive slope of dTm/dp, which was opposite to G-quadruplexes and should be $\Delta K>0$.

Answer: The references have been added. As mentioned in the text, the signs of the quantities refer to relatively low (melting) temperatures (around room temperature), where the slope dTm/dp and ΔK are generally negative. At higher (melting) temperatures (e.g. at a higher salt concentrations), the slope and ΔK become positive, as correctly pointed out by the reviewer. Our analysis also predicts that this happens for duplexes at temperatures around 50°C, and for quadruplexes at much higher temperatures (please see manuscript text on pages 12-16).

2) The authors claimed hydration is the key to the trend of Tm vs pressure. In general, DNA duplexes uptake water molecules when they folds (e.g., J. Phys. Chem. B 1999, 103, 41, 8759–8767), whereas G-quadruplexes release water molecules (e.g., J. Am. Chem. Soc. 2010, 132, 48, 17105–17107). Are all cases valid in the authors’ treatment?

Answer: This is indeed the case and our analysis is in line with this observations. The conclusion that (de)hydration mainly determines the trend of the pressure dependence of Tm follows from the fact that the slope dTm/dp is determined by the volumetric quantities in which the hydration contribution and its temperature dependence are essential. The only basic limitation in our analysis is the postulated linearity. Therefore, the analysis is valid for any (un)folding step of a biomolecule where a linear relationship between Tm and pressure is observed.

3) On page 6, to show the validity of equation 12, it seems that the authors demonstrated using reported values as shown in the sentence “These data suggest…”. The data should be shown in the Supporting information.

Answer: Thanks for the remark. The data has already been listed in Table S1 (now we added the corresponding reference in the text). We have also included the estimated values of the heat capacities in this table. The values of ΔK calculated from eq. 11 and eq. 12 differ by up to 20%, which

is within the experimental error. For the extrapolations, we used a simplified equation (eq. 12) and successfully validated it by comparing the calculated values with the measured ones (Figure 1).

4) The authors' interpretation that the correlation of Tm and pressure is explained as the linear function with the slope derived from the ratio of ΔK and ΔE. Then, how about the physical meaning of the intercept of the linear function?

Answer: The intercept on the ordinate obtained by linear extrapolation of Tm versus p line to zero pressure has no physical meaning. Namely, the linear dependence is determined by the physical properties of the DNA in solution. At a pressure of zero there is no solution - the system is in the vapor phase (ideal gas).

5) The stability of DNA highly depends on the cation binding. However, the authors only discuss hydration, and this study did not include any information about the contribution of the cation binding.

Answer: The release or uptake of cations and water molecules upon DNA (un)folding is included in the quantities of unfolding ($\Delta F = \overline{F_{\mathrm{U}}} - \overline{F_{\mathrm{F}}} + \sum_{\mathrm{j}} n_{\mathrm{j}} \overline{F_{\mathrm{j}}}$, $F = V, E, K, H, C_p$; please see the manuscript text on pages 3 and 6) through the apparent number of exchanged cations or water molecules, n_{j}, and the corresponding partial molar quantity $\overline{F_{\mathrm{j}}}$. The derived equation 12, which links ΔK, ΔE, ΔV and ΔH, is valid for a given reaction stoichiometry (constant n_{j}). This applies If Tm at a given pressure is changed by small addition of salt (e.g. up to 0.5 M NaCl; Tm versus log[Na+] plots are linear, ideal behavior - independence of $\overline{F_{\mathrm{j}}}$ on changing the solution composition can be assumed). At very high salt or osmolyte concentrations the values of n_{j} and $\overline{F_{\mathrm{j}}}$ differ from those in dilute buffer solutions. In addition, n_{j} and $\overline{F_{\mathrm{j}}}$ values differ for different types of cations. Therefore, the linkage equation is only valid if all the parameters ($\Delta K, \Delta E, \Delta V, \Delta H$) refer to a similar solution composition (dilute buffer solutions) and the same cation type. Taken together, the contribution of cation release upon DNA unfolding is an intrinsic component of all the studied thermodynamic parameters of unfolding, that however, cannot be extracted from our analysis.

6) The identification of Tc is interesting. What does Tc mean in physical chemistry and biochemistry of DNA melting? In the G-quadruplex, Tc shows higher than the boiling point of water.

Answer: Thank you for this observation and comment. In line with the suggestion, we have added to the manuscript text (pages 12 - 16) an interpretation of the temperature dependence of volume and compressibility and the pressure dependence of DNA stability that follows from the quantitative analysis presented in a recent interesting article (Makhatadze, Marky and coworkers, Biophys. J. 2022, 121, 4892.). The introduced discussion attempts to clarify the significance of Tc in physical chemistry and biochemistry of DNA melting.

Reviewer: 2

Recommendation: This paper may be publishable, but major revision is needed; I would like to be invited to review any future revision.

Comments:
The analysis presented in this manuscript might be interesting. However, it appears the authors may not be cognizant of a recent publication that establishes a direct correlation between hydration changes and the volumetric properties of dsDNA (Makhatadze GI, Chen CR, Khutsishvili I, Marky LA (2022) The Volume Changes of Unfolding of dsDNA. Biophysical Journal, 121(24):4892-4899. doi: 10.1016/j.bpj.2022.08.005.) This BJ paper also discusses the temperature dependence of the volume changes, and thus the pressure dependence of DNA stability. It would be beneficial for the authors to frame their analysis within the context of this BJ paper, demonstrating what unique perspectives they can offer on DNA stability. . I am open to assessing a revised version. Regrettably, in its current form, the manuscript falls short in a number of categories with the “novelty” aspect being the most critical.

Answer: We thank the reviewer for his/her constructive comments. In our work, we have mainly focused on a related but somewhat different aspect of the volumetric properties of DNA than those discussed in the BJ paper (mentioned by the reviewer), namely the influence of compressibility and expansibility on thermal stability. For this reason, we unfortunately overlooked this interesting BJ paper. The paper is indeed very relevant to our study as it analyzes the volume of DNA (un)folding as a function of temperature. By considering the analyzes presented in the BJ article, we avoided rather general statements about the influence of hydration on the presence of convergence temperature.

We see our analysis as complementary to the BJ article - while the BJ article focuses on the decomposition and calculation of the overall ΔV associated with DNA unfolding, our analysis provides a detailed explanation of how ΔV relates to other thermodynamic quantities such as compressibility and expansibility and their relationship to thermal stability. This has not been previously addressed in the literature - Eq. 12 provides a novel relationship between expansibility and compressibility, while Eqs. 18 - 21, derived from Maxwell's relationship, show how expansibility and compressibility depend on temperature and pressure, and suggest for the first time the existence of a convergence temperature T_c (around 50 °C (duplexes) and around 120 °C (G-quadruplexes)) at which compressibility is zero.

Based on the reviewer’s comment, we have added to the manuscript text (pages 12-16) an interpretation of the temperature dependence of volume and compressibility and the pressure dependence of DNA stability that follows from the analysis presented in the BJ article. We hope that we have gotten to the heart of the matter, and at the same time we are open to further discussion if additional points need to be clarified.
